# IgD lambda plasma cell neoplasm with extensive AL amyloidosis

**DOI:** 10.1002/jha2.234

**Published:** 2021-05-21

**Authors:** Muntadhar Al Moosawi, Julia L. Varghese, Hayley Merkeley, Stephen Parkin, Mohammad Bahmanyar

**Affiliations:** ^1^ Hematological Pathology Department of Pathology and Laboratory Medicine University of British Columbia Vancouver British Columbia Canada; ^2^ Department of Medicine University of British Columbia Vancouver British Columbia Canada; ^3^ Providence Health Care St. Paul's Hospital Vancouver British Columbia Canada; ^4^ Clinical hematologist Vancouver General Hospital Vancouver Canada

A 65‐year‐old male presenting with chest pain and unintentional weight loss was found to have normocytic anemia (hemoglobin of 107 g/L). Endoscopy revealed features of gastritis and biopsies from the stomach and duodenum showed amyloid deposits as demonstrated by histological examination and Congo red staining. Serum protein electrophoresis detected a small band measuring 0.8 g/L at the gamma region which was identified as an IgD lambda monoclonal protein by immunofixation (panel A). Free monoclonal lambda light chains were detected in the urine. Serum lambda to kappa free light chain ratio was 74. There were no lytic lesions on skeletal survey. Both high sensitivity cardiac T troponin and NT‐proBNP were elevated. Troponin was 30 ng/L (normal: <14 ng/L) and NT‐proBNP was 946 ng/L (n: <300 ng/L). Beta 2 microglobulin level was normal (1.5 mg/L). Also, kidney function and calcium level were normal. Bone marrow aspirate and biopsy showed lambda‐restricted plasma cells by flow cytometry immunophenotyping (panel B). A deposition of pink‐blue amorphous and acellular amyloid deposits was detected in the marrow aspirate slides (panel C, original magnification × 50, Wright‐Giemsa). Plasma cells accounted for 30%–40% as highlighted by lambda immunohistochemical stain (panel D, original magnification x20). The trephine biopsy showed thick blood vessels as well as interstitial amyloid deposition that appeared “salmon‐pink” by Congo red stain and showed “apple‐green” birefringence using polarized light (panel E‐J, original magnification x20). The patient was also found to have evidence of multisystem involvement on cardiac MRI and by elevated cholestatic liver enzymes. He has been started on chemotherapy using CyBorD protocol (cyclophosphamide, bortezomib, and dexamethasone) and will be considered for autologous stem cell transplant depending on his response.

Due to the protean presentation of AL amyloidosis, there is often a delay in diagnosis. AL amyloid is a rare disorder secondary to a plasma cell dyscrasia. Furthermore, IgD plasma cell neoplasm is exceedingly rare, and therefore, as this case highlights, a thorough evaluation should be considered in the presence of even a faint IgD band on electrophoresis.


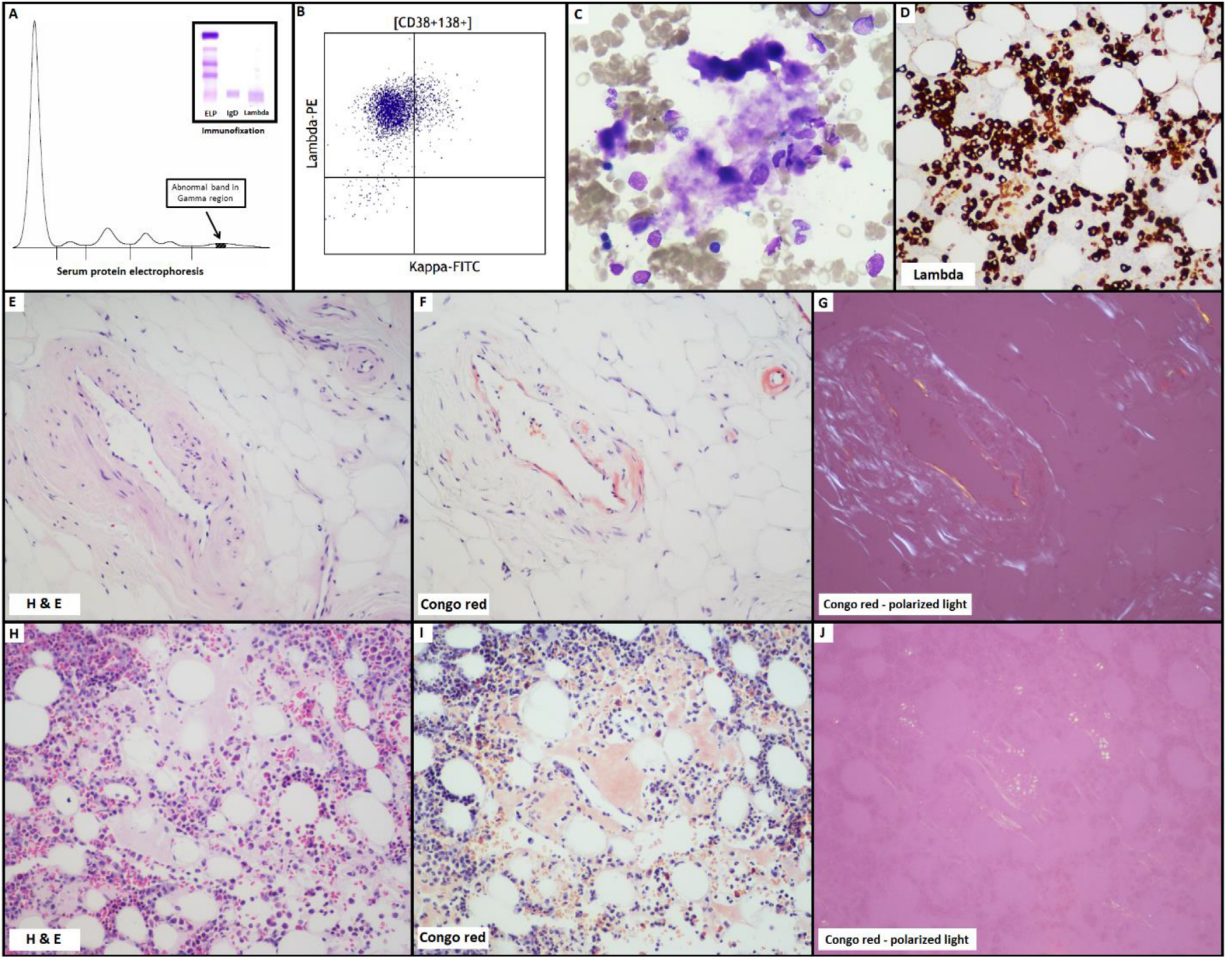


## CONFLICT OF INTEREST

Authors declare no conflict of interest related to this manuscript.

## Data Availability

The data that support the findings of this study are available from the corresponding author upon reasonable request.

